# The pragmatic functions of emojis in Arabic tweets

**DOI:** 10.3389/fpsyg.2022.1059672

**Published:** 2023-02-27

**Authors:** Amjad Alharbi, Mohammad Mahzari

**Affiliations:** Department of English, College of Science and Humanities in Al-Kharj, Prince Sattam Bin Abdulaziz University, Al-Kharj, Saudi Arabia

**Keywords:** emoji, pragmatic functions, hashtags, social media, Arabic

## Abstract

Emojis have been used on different platforms and in different languages to express feelings and emotions in online communication, which has led to their widespread familiarity among social media users worldwide. The current study is concerned with the pragmatic functions (speaker and contextual meanings) of emojis in Arabic tweets. The study used mixed methods to analyze the use of emojis and emoji position to identify their functions and possible gender similarities and differences. The dataset of this study consists of 421 Arabic tweets by Arab users at the beginning of 2022 (149 by male users and 272 by females). This study addressed three questions: (1) What are the common emojis used in Arabic tweets and their position in the tweet? (2) What are the pragmatic functions of these emojis? (3) What are the possible differences and similarities between male and female users in the use of emojis? The findings revealed that *Loudly Crying Face*, *Red Heart*, *Face with Tears of Joy*, *Broken Heart*, *Smiling Face with Heart-Eyes*, *Pleading Face*, *Slightly Smiling Face*, *Pensive Face*, and *Weary Face* were preponderant in Arabic tweets. Also, this study found seven pragmatic functions ranging from the most to the least frequent: *Multiple functions*, *Reaction*, *Action*, *Decoration*, *Physical action*, *Softening*, and *Tone modification*. Regarding the role of gender, there were both similarities and differences in terms of the frequency of emoji use, emoji position, and function. Moreover, the findings showed the importance of context to interpreting the functions of emojis. Finally, the findings have implications for emoji designers and Natural Language Processing (NLP).

## 1. Introduction

Communication is essential to people; they use words to communicate their feelings and thoughts, which refer to verbal communication. However, the new generation currently tends to use emoticons more than words or sentences as a means to express their feelings, such as happiness or sadness, which refer to non-verbal communication. Word use has decreased as sign language and emoticons have increased in use. According to Pavalanathan and Eisenstein (2015) that emojis are “picture characters” which include not only faces, but also concepts and ideas, such as weather, vehicles and buildings, food and drink, or activities (e.g., running and dancing). However, emoticons are the expressions of emotions that mimic non-verbal cues in speech (Rezabek and Cochenour, 1998; Wolf, 2000; Crystal, 2006).

Recently, emojis have become available on any form of electronic device because they are conversational, full of meaning, and strengthen the tone and closeness between participants, resulting in their widespread use among social media users (Danesi, 2017). According to Danesi (2017), the graphic system generally includes emoticons, emojis, stickers, images, videos, and so on, which help the users to communicate their feelings in online communication. As mentioned by Hymes (1972), using a language requires linguistic competence and communicative competence as well as knowing the rules of the language and how to use these rules to communicate. The same applies to using emojis, which requires communicative competence and knowing how to use the emoji in the appropriate way, as well as facility in switching from alphabetic writing to emoji writing, which is another feature of emojis subsumed under code switching (Danesi, 2017). Consequently, Internet users must be competent in choosing and combining emojis with messages to be able to write meaningful messages that maintain the relations between the users and indicate their positive sides (Danesi, 2017). The correct use of emojis is thus essential for effective conversation (Imran et al., 2020). For example, when someone sends a message with a happy face to his boss when they do not have a close relationship, it is seen as unprofessional. On the other hand, if they have a close relationship, it is seen as a friendly tone. Sending a message without an emoji cannot be seen as a complete thing and shows that the sender is not interested in the conversation too (Imran et al., 2020). In addition, the interpretation of emojis is important because failing to interpret emojis correctly may lead to misunderstanding and miscommunication between people (Imran et al., 2020). Danesi (2017) mentioned that one participants reported that he made his comments pleasant by adding a smiley face in them, even when the news was bad, and at the same time it gave him a sense of belonging to the person to whom he was writing. Further, there are some conditions for the use of emojis, such as the sender’s mood at the moment of sending and a close relationship with the receiver (Konrad et al., 2020).

Emojis can be used in many contexts including, work, study, chatting, discussion, and even advertising. For instance, emojis can be used by teachers to provide visual feedback to their students. Also, students use emojis in their study groups. Emojis are used in chatting either private or public as well as in online discussion platforms to discuss the last issues. Lastly, emojis are used by brands to attract the consumers’ attention (Romig, 2015; Sampietro, 2019; Alshboul and Rababah, 2021; Etman and Elkareh, 2021; Cavalheiro et al., 2022).

A single emoji may mean thousands of words, which is why the graphic system is not considered a universal language (Herring and Dainas, 2018). There are many functions of emojis and people vary in their uses and interpretations regarding emojis (Miller et al., 2016, 2017). Variations in interpreting emojis are due to a number of reasons, such as the familiarity between the sender and the receiver, the gender of the receiver, and the culture in which the emoji is utilized. For example, the pizza emoji is frequently used in American culture, the love emoji is frequently used in French culture, and the rose emoji is frequently used in Arabic culture (Miller et al., 2016; Tigwell and Flatla, 2016). Broadly speaking, emojis are used by various cultures, age groups, and genders as means to express a variety of feelings and thoughts just by clicking a button. One emoji may have more than one meaning in online communication as people interpret the emojis in different ways, and the meaning largely depends on the context where the emoji is used (Al Rashdi, 2018; Alshboul and Rababah, 2021; Dainas and Herring, 2021). Emojis are treated as an autonomous language by many language scholars, as they can be used without any textual accompaniment to replace the words (Dainas and Herring, 2021).

Computer-mediated communication (CMC) refers to “the process by which people create, exchange, and perceive information using networked telecommunications systems that facilitate encoding, transmitting, and decoding messages” (Romiszowski and Mason, 2013, p. 398). Computer-mediated discourse (CMD) is the communication produced when human beings interact with one another by transmitting messages *via* networked or mobile computers, where ‘computers’ are defined broadly to include any digital communication device. CMD is a specialization within the broader interdisciplinary study of computer-mediated communication (CMC) which is distinguished by its focus on language and language use and the use of methods of discourse analysis to address that focus” (Herring and Androutsopoulos, 2015, p. 127). Social media platforms provide a perspective on social interaction that enables researchers and linguists to understand social networking and study language use on the Internet.

Twitter is a form of database that contains a large number of tweets and links, as well as providing information about the users and the total numbers of their tweets and followers. The Twitter platform has been compared to Google Search as a useful website to collect the information, to negotiate meaning, and maintain relations. “Hashtags are an emergent convention for labelling the topic of a micropost and a form of metadata incorporated into posts” (Zappavigna, 2012, p. 1). It can thus be said that hashtags are a common way to discover trendy topics and issues in social media, which is different from websites that rely on one type of interaction, such as question and answering websites (Scardigno and Mininni, 2020). Recently, Twitter has become one of the most common social media networks used in Arabic countries (Blogger, 2021). Tankovska (2021) stated that Saudi Arabia was one of the top countries in Twitter users in January 2021, where Twitter was used mainly to communicate with other people, exchange information, and find out about “hot” public issues.

A number of studies of emojis has been conducted regarding their use in languages like Chinese (Herring and Ge, 2020), Turkish (Yüce et al., 2021), Indonesian (Muzakky et al., 2021), English (Tantawi and Rosson, 2019; Konrad et al., 2020), and Spanish (Sampietro, 2019). However, there have been few such studies of Arabic. In addition, studies of emojis in Arabic have focused on certain platforms, such as WhatsApp, Facebook, and Telegram, but few have focused on Twitter, even though different platforms may render different emojis with different functions as will be explained widely in the literature review. Hence, the current study seeks a linguistic understanding of emoji use in naturally occurring Arabic tweets. The purpose of the current study is to identify the most common emojis used in Arabic tweets, analyze their functions, and identify possible differences in emoji use between male and female users. This study will shed light upon the functions of emojis in Arabic tweets, and seeks to draw linguists’ attention to one of the most important topics for research within the next few years. This study is significant because it will help people to understand the importance of context to interpret the meaning of emojis in our daily communication.

## 2. Emojis

The word “emoji” derives from a Japanese word formed from *e*, meaning “picture,” and *moji* meaning “letter.” Consequently, emoji can be defined as a picture-word; it can be used as singular or plural in English (Danesi, 2017). *Merriam-Webster Dictionary* defines emoji as “any of various small images, symbols, or icons used in text fields in electronic communication (as in text messages, email, and social media) to express the emotional attitude of the writer, convey information succinctly, communicate a message playfully without using words, etc” (Emoji, 2021).

The first use of emoji started with Scott Fahlman in 1982 when he used a smiley face “: -)” at the end of the sentence in an e-mail message to indicate a positive mood. Then, in the late 20^th^ century a typical collection of emojis was made by Shigetaka Kurita through the use of Japanese mangas and kanji (Blagdon, 2013). In the beginning, there were only 176 emojis, whereas by 2019 the entire number of emojis had reached 3,019 (Wirza et al., 2019). As a result of the growth of technology, a larger number of emojis have been made with different emoji faces and different skin tones, and a dictionary has been created specifically of emojis and their meanings; for example, the *Oxford English Dictionary* selected the emoji of a face with tears of joy as the trendy word in 2015 (Kramer, 2015). Furthermore, Apple released the IOS5 system, which included emojis, as a step toward increasing the popularity of emojis and emphasizing their power in 2010. Also, emojis have been unified in the Unicode standard form, allowing them to be utilized in practically all languages and applications around the world (Danesi, 2017; Ge and Herring, 2018; Konrad et al., 2020; Abbas and Ubeid, 2021; Al-Garaady and Mahyoob, 2021). Emojis were created to make emoticons more comprehensive in pictures; however, both emojis and emoticons have similar functions such as tone marking (Konrad et al., 2020).

Recently, the use of alphabetic writing has been somewhat supplanted by emojis in how users communicate with each other in online communication (Imran et al., 2020). Some problems appeared when people wanted to share their ideas or feelings in the virtual environment because of the absence of facial expressions, gestures, intonation, and prosody that help to convey the intended meaning. However, CMC solved this problem by enabling social media users to use a great variety of emojis as a means to express their emotions and feelings, including happiness, sadness, and anger. Thus, emojis provide various gestures as means to enhance online communication and make it to some extent similar to face-to-face communication and thereby enhance its position as an alternative to it (Dolot and Opina, 2021). Emojis serve to make clear the real meaning of the written message. For example, using the face-with-tears-of-joy emoji after a joke signals laughter or humor. According to Lo (2008), a message with a smiley face conveys a positive meaning, in contrast to a message without a smiley face. In addition, emojis provide various types of faces with different facial expressions, resulting in several other interpretations.

According to Na’aman et al. (2017), emojis could have different functions, such as content words or indicated particular attitudes. Herring and Dainas (2017) indicated that there were several functions of emojis, such as reaction, tone, action, mention, riff, and narrative sequence. Moreover, Al Rashdi (2018) found that emojis indicated the tones of the messages, created alignments among group members, and created interactive communication. Similarly, Gibson et al. (2018) stated that the “face covering hand” emoji served to indicate laughter and ironic in the message. As mentioned by Danesi (2017), emojis were used to perform phatic functions, such as opening and ending a conversation and avoiding silence. More generally, emojis were used to perform emotive functions, such as expressing opinions and attitudes or sentiment, that were essential for online communication. In addition, while 90% of respondents agreed in their interpretation of the face-with-tears-of-joy emoji, the interpretations of the least frequent emojis differed based on the context and the relation between the sender and receiver (Dainas and Herring, 2021).

As mentioned by the previous studies, there are many functions for the emojis. However, emoji position in the sentence plays an important role in identifying the function of the emoji (Arafah and Hasyim, 2019a,b; Park and El Mimouni, 2020; Etman and Elkareh, 2021). For example, if the emoji is positioned at the beginning of the sentence, then it functions to start the sentence. However, if the emoji is positioned at the end of the sentence, it functions to close the sentence and to replace the punctuation marks.

According to Doiron (2018), emojis became an important part of the lexicon because most people are able to recognize and use them routinely in their daily interaction as means to disambiguate meaning. As Romig (2015) stated, emojis can be used to make comments stickier instead of the traditional way of commenting and to provide visual feedback for students. For example, a face with sunglasses indicates that a piece of work is cool. It is thus clear that emojis have different pragmatic functions in social media platforms and thus merit closer scholarly consideration.

## 3. Literature review

This section discusses previous studies that touch on the functions of emojis in different platforms of social media, such as Twitter, Facebook, WhatsApp, Telegram, and so on, either in Arabic or other languages. Although early studies on emojis proposed that they have similar functions as emoticons, emojis are more multifunctional. As mentioned by Dainas and Herring (2021), there were many reasons which lead to different functions for emojis, such as the context and different platforms render emojis in a different way. That is why emoji is more open to several interpretations. Also, social and cultural factors affect how social media users interpret emojis. Therefore, there is a suggestion that each emoji functions pragmatically in online communication (Herring and Dainas, 2017).

On Twitter, Tantawi and Rosson (2019) investigated the paralinguistic function of emojis in the United States based on data collected from Twitter messages. The findings showed that 43% of emojis had the paralinguistic feature of attitudes, such as playfulness, praise, and confusion. Also, the face with tears of joy and loudly crying face emojis were frequently used by American users. In terms of topic analysis, it was found that “society” was a common topic, showing that Twitter is used for discussing recent issues in society, in contrast to other platforms. Therefore, this study provides evidence that Twitter is typically used to discuss the latest trends in society. In addition, Al-Rawi et al. (2020) explored the effect of gender on emoji usage during the COVID pandemic. The data were collected from Twitter hashtags related to COVID-19, from which 600 emojis were collected and analyzed to understand the sentiment of each emoji. The findings showed that men used emojis in a positive way, such as the folded hand emoji and global emoji to show solidarity. In contrast, women used the emojis negatively, such as the broken heart emoji. In addition, some emojis were related to topics during the pandemic like health care, mortality, and financial and employment issues. However, the findings cannot be linked to a certain country because the tweets were collected from several countries. Additionally, Yüce et al. (2021) examined the functions of emoji in Turkish. The data were collected from Turkish tweets by sport clubs, and the emojis were analyzed using content analysis. The findings showed that the emojis used by sport clubs denote the colors and symbols related to their clubs, such as the lion emoji that is Galatasary club’s symbol and the eagle emoji that is Besiktas club’s symbol. The importance of using emojis can help sport clubs in their marketing communication goals in creating loyalty that need to be conveyed on individuals effectively. Although the emojis in their study indicated positive meanings, their study was limited in that emojis were analyzed without context and might have different meanings depending on the context. Etman and Elkareh (2021) examined the use of emojis across Arabic dialects including Saudi, Egyptian, Kuwaiti, Emirati, Iraqi, and Lebanese. The data were collected from Arabic tweets and included numerous emoji for facial expressions and hand gestures. The findings showed that Arab users use emojis to express their facial expressions. In addition, the face with tears of joy was the most common emoji across Arabic dialects and is used to indicate laughing then loudly crying. However, their study differs from the current study as follows: Their study aimed to discover the alternative ways Arabs use emoji across different dialects to compensate for the absence of a non-verbal component, while the aim of the current study was to identify the pragmatic functions of emojis. Also, they analyzed the data with the help of custom Python scripts, while the current study analyzes the data based on taxonomy of Herring and Dainas (2017). However, the other studies on emojis on Arabic were on different platforms.

For instance, Mahzari (2017) identified the verbal and non-verbal strategies of expressing congratulations on Facebook. The data of his study were collected from Facebook comments by Saudi users, and the findings showed that Saudi users used certain strategies for congratulations, such as congratulations, offering wishes, praise, and so on. These strategies were used with emojis such as the red rose, slightly smiling face, red heart, and bouquet of flowers, which were the emojis most used by Facebook users. In addition, these emojis were categorized into seven functions; however, expressing endearment was the most frequent function in the data. The researcher suggested that additional studies need to be conducted on other platforms, such as WhatsApp, Twitter, and Text message, to better understand the functions of emoji in different contexts. Also, Alshboul and Rababah (2021) analyzed the functions of emojis in Jordan. The data were collected from a Facebook group with 400 members. The researchers built a corpus of emoji to analyze their functions. Also, they administered a questionnaire as a means to identify any differences in emoji use between male and female users. The findings revealed that emojis were used to indicate emotive functions, conative functions, phatic functions, poetic functions, referential functions, and metalingual functions. In addition, they found that women used emojis to share their feelings more than men. The researchers pointed out that there is a need to analyze the functions of emojis in other social media platforms. In addition, Herring and Dainas (2020) assessed gender and age influences on the interpretation of emoji functions among English speakers based on data collected from public groups on Facebook whose members differed by gender and age. The findings showed that gender does not affect the interpretation of emojis, but age does. The younger users interpreted the emoji as softening or tone, while older users interpreted the emoji as action. Thus, emoji use is not defined by gender, as male and female interpretations of emoji are similar, differing only in their proportions of use. Moreover, Dolot and Opina (2021) investigated the functions of graphicons in Filipino using data collected from chats in Facebook groups and analyzed based on Herring and Dainas’s model of graphic functions (Herring and Dainas, 2017). The findings showed that Filipino users used emojis more than stickers. Also, five additional functions beside those of Herring and Dainas’s model were discovered: response, replacement, sharing, complement, and attention. The findings of this study hold implications for future research on all graphicons in a message as well as confirming the value of concentrating on private messages on other platforms. So far, response and sharing are used on certain platforms like WhatsApp or Facebook due to the nature of these platforms as places where users share their photos or answer questions, unlike other platforms.

On WhatsApp, Al Rashdi (2018) examined the functions of emojis in Oman. The data were collected from WhatsApp, with 15 male respondents and 30 female ones. The findings showed that there are seven functions of emojis in Omani WhatsApp. For instance, emojis were used to signal emoticons, to signal celebrities, to signal approval, to signal the opening and close of the conversation, to serve as linking devices and as contextualization cues, and finally for use as a response to compliments. However, some distinct emojis in the data were used for the same function. For example, a face blowing a kiss and a thumbs-up sign were used to indicate approval. Therefore, the results demonstrated that emojis were not only used as indicators of language users’ emotions, but also they worked for many other communicative functions. As the researcher mentioned, additional studies should be done to examine emojis in mixed WhatsApp groups, as the surrounding text is crucial to understand the function of the emoji. Also, Sampietro (2019) explored the functions of emoji in Spanish by focusing on three domains: the illocutionary, discourse, and stylistic domains. The data were collected from WhatsApp chats from the researcher’s family and friends and included 1,077 emojis. The results showed that emojis are used to foster relations between people, as in the smiling face with smiling eyes; serve to open and end the conversation, as in the face blowing a kiss; and are used in informal groups and contexts, as in a grimacing face and the face with tears of joy. As the researcher noted, more work is needed on formal groups to explore the functions of emoji more thoroughly. Nevertheless, the face blowing a kiss was found in this study to be used to open and end conversations, as it is customary on WhatsApp to open and end chats. However, it may be also used for other purposes on other platforms. Further, Muzakky et al. (2021) examined the pragmatic function of the folded hand emoji among Indonesians. The data were collected from four lecturing WhatsApp groups and screenshots of the messages. The findings showed that the folded hand emoji is not used only to express emotions, but serves the three pragmatic functions of thanking, apology, and request. It has been suggested that researchers explore gender effects and interview users to better understand their specific purposes for using it.

On Telegram, Abbas and Ubeid (2021) explored the connection of emojis to the surrounding messages and how they affect the interpretation of meaning by collecting data from a chat group on Telegram used by Iraqi students as data to analyze the frequency and functions of the emojis used. The findings showed that the emojis were used to indicate parallel emotions, attitudes, and humor, enhance emotion intensity, express irony, modify illocutionary force, and enhance attitude intensity. Their study revealed that there is a relationship between emojis and text, and emojis were used to communicate facial expressions as well as body language as in face-to-face communication. In addition, Ge and Herring (2018) analyzed whether an emoji sequence functions like an utterance. The data were collected from messages on a Chinese website, Sina Weibo, by 87 celebrities. The findings showed that the emoji sequences functioned as verbal utterances containing verbs, adjectives, and pronouns with an OVS word order. Also, the users used these emoji sequences to express actions and activities, such as a smiling face with heart eyes, dancing, and women holding hands. In addition, the findings indicate that emojis in Sina Weibo website have syntactic properties related to those of the Chinese language. This study offers some important insights into the syntactic properties of emojis in the Chinese language and proposes that emojis have a syntax.

Different tools such as interview and survey have been used to investigate the use of emojis in data collection. For example, Herring and Dainas (2018) investigated whether gender affects the use and interpretation of emojis. The data were collected through a survey assessing how male and female respondents interpreted the emojis. A comment with an emoji in a Facebook group was included in the survey, and respondents were asked to choose the function of the emoji. The findings showed that female users used emojis more than males: 92% of female users in contrast to 78% of males. Also, there was no difference between men and women in their interpretations of the emojis, as they were largely in agreement. Also, Imran et al. (2020) studied the gender differences in using emoticons based on data collected through an online questionnaire completed by 75 participants, whose gender, age, and qualifications were elicited. The findings showed that men tend to use emoticons as means to replace words more than women. In addition, where male users used emojis to emphasize meaning, female users used emojis to express their feelings. Also, most of the participants interpreted the emotions easily because emoticons allow the users to express their feelings and emphasize the meaning in online communication, unlike words alone. In addition, Al-Garaady and Mahyoob (2021) investigated the functions of emojis in social media applications in Saudi Arabia based on a survey-based questionnaire given to 143 students at Taibah University. The findings showed that female users used emojis more than male ones. The most common emojis were the face with tears of joy, followed by the smiling face with heart eyes, pleading face, loudly crying face, red heart, and finally thumbs-up emoji. However, the face with tears of joy was used by both males and females, in contrast to the other emoji, which were used more by female users. As the researchers stated, learners have a tendency to use emojis instead of words to avoid spelling mistakes and to connect the language with the meaning.

Likewise, Wirza et al. (2019) investigated the effects of gender on emoji use in Indonesia based on data collected by interviews with 20 male and 20 female university students and messages with emojis, finding a slight difference between male and female students in emoji choice: Where the males used the folded hand emoji more than the females, they in turn used the loudly crying face more than males. Gender influenced emoji choice as well as the number of emoji used in the messages, but it does not entirely determine emoji use, as both used the face with tears of joy emoji to communicate their feelings in online communication. Also, Konrad et al. (2020) examined how emoji and stickers are used by English speakers. The data were collected using semi-structured interviews and a large-scale survey to understand emoji and sticker use. The findings show that emojis are used for expressing emotions. However, stickers are more intense than emojis and are used in very close relationships, in contrast to emojis. It has been mentioned that further research should be done to explore emoji and stickers on other platforms and to compare Asian and Western usage.

The previous studies showed the similarities and differences in terms of the use and functions of emojis across the different platforms of social media. However, Twitter hashtags that include trendy topics in social media need more studies to explore how Twitter users use emojis and their functions in the tweets on trendy hashtags. Therefore, the current study seeks to identify the most common emojis used in Arabic tweets, analyze their functions and positions, and identify possible differences in emoji use between male and female users.

## 4. Research methodology

### 4.1. Aims and research questions

The main goal of the current study is to analyze the use of emojis and emoji position to identify their functions and possible gender similarities and differences. The research questions are:

1. What are the common emojis used in Arabic tweets and their position in the tweet?
2. What are the pragmatic functions of these emojis?
3. What are the possible differences and similarities between male and female users in the use of emojis?

### 4.2. Data collection

The data of this study were collected from Twitter, because it is widely used by Arabs. Blogger (2021) noted that there were 27.08 million social media users in Saudi Arabia in 2021, 71.40% of whom use Twitter. Since this study focuses on functions of emojis in Arabic tweets, only Arabic tweets with emojis were collected. The researchers manually collected only the Arabic tweets with emojis because this study focuses on emojis within context in order to identify their functions. Therefore, the researchers excluded the tweets that have only text, only emojis, language other than Arabic, or the gender of tweeter was not clear. The gender was specified based on the person’s name on the profile, profile description, and the masculine or feminine marker of tweeter in the Arabic words in the tweets. In addition, Arabic tweets from 29 various Arabic hashtags were collected in order to gather a variety of emojis. The selected hashtags included topics popular at that time in Saudi Arabia like The Boulevard, Friday Day, National Day of Saudi Arabia, final exams, Salam Mall Movie, and Chelsea vs. Liverpool. Those hashtags were selected for this study because they were active at the time of data collection. With regard to time, data collection took place at the beginning of 2022. A total of 421 tweets (272 and 149 by female and male users, respectively) randomly by using manual selection, with a total number of words of 2,853. In all, 102 distinct emojis were collected with their context from Arabic tweets, with 439 emoji collected total; however, repeated emojis were treated as one when counting frequency. The researchers used Excel sheets to help them in counting the frequency of emojis and functions. For instance, Loudly Crying Face emoji was used 31 times in the corpus; however, the repeated emoji was counted as one when they appear in the same tweet and have the same function.

### 4.3. Data analysis

The emojis were analyzed with the help of Herring and Dainas’s (2017) taxonomy, which provides the functions and formal definitions of all types of graphicons. Since this study focuses on the functions of emojis, their taxonomy suits the functions of emojis found in Twitter. In other words, Herring and Dainas proposed a taxonomy for the functions of graphicons in 2017, then in a study in 2018, they asked participants to choose the function of an emoji from a list to test the taxonomy. The findings showed that the participants agreed with the researchers’ interpretation. Also, while other models fit certain platforms like WhatsApp or Telegram, they were not suited to the Twitter platform. For example, in Twitter, there is no opening or ending conversation. Therefore, we analyzed the emojis based on Herring and Dainas’s taxonomy because they interpreted the functions of emojis not only from their perspective as researchers, but also from the perspective of participants, which could validate their taxonomy. In the current study, the researchers are native speakers of Arabic, and they are from Saudi Arabia. Therefore, their linguistic and cultural background helped them to understand and interpret the functions of emojis based on the context of Arabic tweets. Reliability was achieved by the following procedures. The first researcher analyzed the pragmatic functions of the first 50 Arabic tweets. The function of each emoji was revised by the second researcher, and then the discrepancies were resolved through discussion. The same procedure in terms of analyzing, revising, and solving the discrepancies was followed for the rest of tweets.

The data were analyzed qualitatively and quantitatively. Mixed-methods research is defined by Loewen and Plonsky (2016) as “Research that combines different paradigms and research traditions in an effort to arrive at a more complete understanding of the object under investigation. Most often mixed methods refers to the combination of qualitative and quantitative research methods” (p. 117). In the qualitative analysis section, the researchers used some tweets from the data as means to show the emojis and their functions in Arabic tweets, based on the context. It is worth mentioning that Arabic is written and read from right to left, unlike English. However, there are some spelling mistakes in some tweets in the data, which were left intact. However, the personal information was omitted from the examples shown to protect the privacy of the Tweeter users. In the quantitative analysis section, the researchers discussed the frequency of emojis and functions in Arabic tweets. Also, a comparison between genders was made in order to show the similarities and differences in emoji use and emoji function. As a way to preserve the data during online communication, the tweets were copied in text format and pasted into a Microsoft Excel document, which allowed the frequency of each emoji type and function to be counted. That is by adding the emoji in the search box in the document, the frequencies as well as the functions of the emoji could be obtained (Table 1).

**Table 1:** Formal and lay descriptions of the pragmatic functions.

Function	Formal description	Example
Tone modification	“Graphicon directly modifies text, clarifying how a message should be interpreted”	I just realized I have a calculus exam Tomorrow 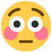
Softening	“Making the comment less forceful or more polite.”	Please help me! 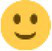
Reaction	“Graphicon used to portray a specific emotion in response to something that has been posted”	[In response to a photo of an attractive celebrity] 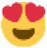
Action	“Graphicon used to portray a specific physical action”	Ha ha, you missed 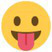
Mention	“Mentioning a graphicon rather than using it”	Sending kisses 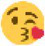
Riff	“Graphicon is a humorous elaboration on, play on, or parody of a previous graphicon or comment”	-
Sequence	“A series of consecutive graphicons (often of the same type) that convey a narrative of some kind as opposed to a composite message”	-
Physical action	“literally doing what the emoji expresses while typing their comments”	This is me right now 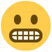
Decoration	“The emoji has no function except to make the text more visually interesting or appealing”	That makes me happy 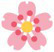
Multiple functions	“The graphicon has multiple, distinct meanings”	[In response to a prompt asking “what makes you happy” alongside an image of a mug with a yellow smiley face]: Like this smiley mug 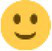 [tone and mention]

Formal and lay descriptions of the pragmatic functions (Herring and Dainas, 2017).

## 5. Results

### 5.1. The pragmatic functions of emojis in Arabic tweets

1. **Tone modification**

Example 1: 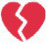 يمه

Transliteration: īummah 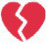


Translation: Mother 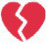


Example 1 consists of a short tweet from a female user taken from a hashtag “word that you love,” and the user mentions mother. In this tweet, it can be seen that the broken heart emoji (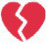) changed the whole tone of the tweet it accompanied. Despite the fact that the user said mother, the tone of this word was completely changed from positive to negative through the broken heart emoji to add sadness feeling to her tweet.

Example 2: 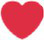 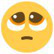 اشعار من شخص تحبه

Transliteration: aišʿār min šaḫṣ tuḥibuh 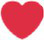 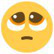


Translation: A notification from someone you love him 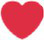 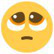


Similarly, Example 2 consists of a tweet from a male user taken from a hashtag “what is the most beautiful thing in life,” and it stands for receiving a notification from a person that you love. In this tweet, it can be noted that the use of the pleading face emoji (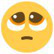) changed the whole tone of the tweet it accompanied. Although the user said a beloved person, the pleading face emoji changed the tone of the tweet from positive to negative, expressing sadness.

2. **Softening**

Example 3: 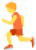 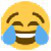 الفلوس لحد يضحك عليكم ويقول غيرها

Transliteration: alfulūs laḥad īḍḥak ʿalaīkum ūī yqūl ġairha 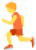 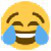


Translation: Only money, no one laughs at you and says something else 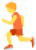 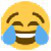


Example 3 consists of a tweet from a female user with the hashtag “what is the most beautiful thing in life”; she reports that money is the best thing in life, whereas other users mentioned such things as family, achieving one’s dreams, and smiling. The user mentioned money and hence used the face with tears of joy emoji (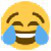) to lighten and soften her comment to be acceptable to others.

Example 4: 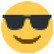 أحتاج قهوووووه

Transliteration: aḥtagˇ qahūūūūūah 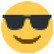


Translation: I need coffee 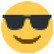


The previous tweet shows the use of emoji to soften a request in Arabic tweets, not only to soften a comment as in Example 3. Similarly, Example 4 consists of a short tweet from a male user in which he mentions that he needs coffee. However, it can be seen that the smiling face with sunglasses emoji (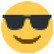) was used to soften his request and make it more courteous.

3. **Reaction**

Example 5: 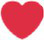 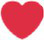 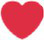 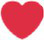 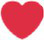 العائله

Transliteration: alʿā ʾylah 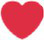 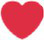 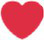 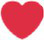 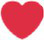


Translation: The family 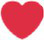 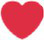 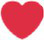 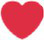 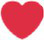


Example 5 consists of a short tweet from a female user taken from a hashtag “what is the most beautiful thing in life,” to which she replies “the family.” Based on the nature of the hashtag topic, the user reacted to her comment using the red heart emoji (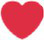) indicating her emotions in response to the comment itself. However, it can be noted here the red heart emoji was repeated in this tweet more than once to emphasize her emotions.

Example 6: لو اشوف المقطع ذا الف مره ما اطفش ههههههههههههههههههههههه 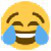 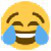 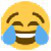 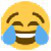 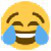 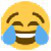


Transliteration: lau ašūf almaqṭaʿ da alf marrah ma aṭfaš Hhhhhhhhhhhhhhhhhhhhhhhh 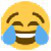 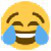 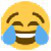 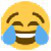 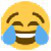 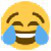


Translation: even if I watch this video a thousand times, I will not get bored hhhhhhhhhhhhhhhhhhhhhhh 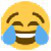 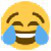 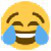 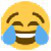 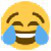 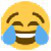


This tweet shows the use of emoji to react to videos or pictures previously posted, and not necessarily reacting to the comment itself as in Example 5. In Example 6, a male user reacted to a posted video using the face with tears of joy emoji (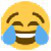) indicating his reaction to the video, not the comment. However, the face with tears of joy was repeated several times to express a more intense reaction.

4. **Action**

Example 7: 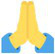 دعواتكم اجلد اختبار اليوم

Transliteration: daʿawatukum agˇlīd iḫtibār alyūm 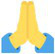


Translation: Your prayers for me to do well in the exam today 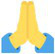


Example 7 is a short tweet from a male user, in which he asks the other users in the hashtag to supplicate for him because he has an exam. The user used the folded hand emoji (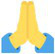) to indicate the action of supplication. Thus, the user here tries to simulate the action of supplication virtually by using the folded hand emoji.

Example 8: 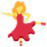 توني استوعب انه اليوم الخميس

Transliteration: taūinī astaūʿib innah alyaum alḫamīs 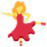


Translation: I just realized that today is Thursday 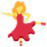


Similarly, Example 8 consists of a tweet from a female user taken from a hashtag “Thursday,” in which the user just realizes that today is Thursday. The use of the woman dancing emoji (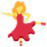) indicates the action of dancing, alluding virtually to celebration.

5. **Physical action**

Example 9: 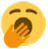 خمول

Transliteration: ḫumūl 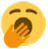


Translation: Laziness 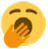


Example 9 consists of a short tweet from a female user taken from a hashtag “what is your feeling now,” and the user mentions that she feels laziness. In this tweet, it can be seen that the yawning face emoji (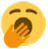) indicates laziness and that the user felt actually what the emoji expresses when the comment was written.

Example 10: 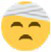

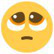
 صدااااع

Transliteration: ṣudāʿ 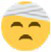

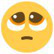


Translation: Headache 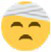

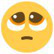


Similarly, Example 10 consists of a tweet from a female user taken from a hashtag “what is your feeling now” and the user mentions that she feels he has a headache. In this tweet, the face with head-bandage emoji (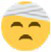) indicates a headache; again the user felt exactly what the emoji expresses when the comment was written.

6. **Decoration**

Example 11: 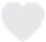 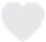 الحمدالله

Transliteration: alḥamdulillah 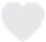 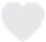


Translation: Thank God 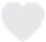 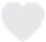


Example 11 consists of a short tweet from a female user, and the user mentions *ālḥamdulillah* ‘Thank God.’ In this tweet, it can be seen that the user used the white heart emoji (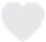) twice as decoration to add aesthetic value to her comment.

Example 12: 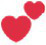 صباحكم جميل

Transliteration: ṣabaḥukum gˇamīl 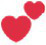


Translation: Have a beautiful morning 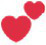


Likewise, Example 12 consists of a tweet from a female user, who wishes a beautiful morning to all. In this tweet, it can be noted that the two hearts emoji (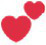) serves no purpose other than to make the text more visually interesting.

7. **Multiple functions**

Example 13: 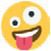 أجمل مباراة في الدوري الإنجليزي

Transliteration: agˇmal mubarah fī alddawrī alinglīzī 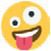


Translation: The best match in the English Premier League 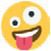


Example 13 consists of a tweet from a male user taken from a hashtag “Chelsea vs. Liverpool,” and the user mentions that it is the best match. In this tweet, it can be seen that the zany face emoji (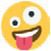) has two possible functions. It can be used for a reaction, that is, to indicate his response to the comment. Alternatively, it can be used for an action, whereby the user here tries to perform a virtual action.

Example 14: 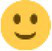 اكره هالاغنيه

Transliteration: akrah ha alauġniyah 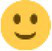


Translation: I hate this song 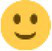


Likewise, Example 14 consists of a short tweet from a male user in which he mentions that he does not like the song. In this tweet, the slightly smiling face emoji (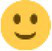) has three possible functions. It can be used for his reaction to a posted video; to soften his criticism; or as an action, here that of smiling virtually.

### 5.2. The frequency of emojis, emoji positions, and pragmatic functions in Arabic tweets

This section includes 11 figures showing the frequencies of emojis, the frequencies of their functions, a breakdown of the emoji for each function, the positions of emojis in tweets, and a summary of gender differences.

**Figure 1:**
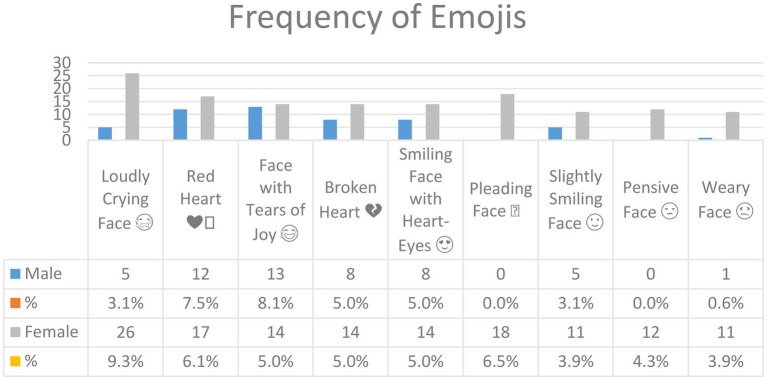
The most common emojis in Arabic tweets.

Figure 1 shows the most common emojis used by Arab users and their frequencies. Whereas the total number of collected emojis was 102, only emojis whose frequency is 10 or more were included in Figure 1 (see Appendix A for those with lower frequency). There are nine emojis in Figure 1 above; another 93 were used fewer than 10 times. However, it is clear from Figure 1 that the *Loudly Crying Face* was used the most in Arabic tweets corpus, 5 times by male users and 26 times by females, followed by *Red Heart* (male, 12; female, 17), *Face with Tears of Joy* (male, 13; female, 14), *Broken Heart* and *Smiling Face with Heart-Eyes* (male, 8; female 14), *Pleading Face* (male, 0; female, 18), *Slightly Smiling Face* (male, 5; female, 11), *Pensive Face* (male 0; female, 12), and *Weary Face* (male, 1; female, 11).

**Figure 2:**
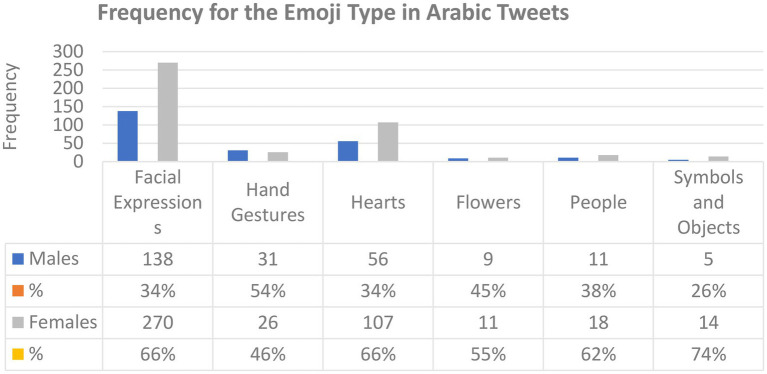
The frequency of use by emoji type in Arabic tweets.

In other words, *Loudly Crying Face* (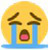) is the top emoji used by female users, *Face with Tears of Joy* (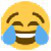) the top emoji used by males. In addition, *Red Heart* (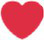) is the third most common emoji among female users and the second most common males. However, there were some emojis that were used only by male users, including 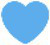, 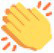, 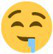, 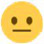, 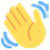, 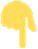, 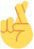, 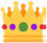, 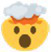, 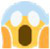, 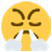, 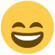, 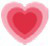, 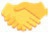, 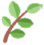, 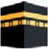, 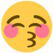, 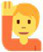, 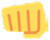, 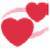, 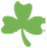, 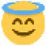, 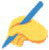, and 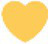. Similarly, some emojis were only used by female users, including 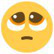, 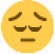, 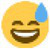, 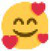, 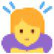, 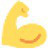, 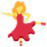, 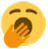, 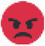, 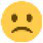, 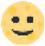, 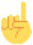, 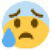, 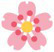, 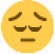, 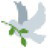, 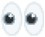, 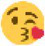, 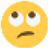, 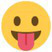, 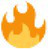, 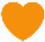, 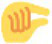, 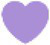, 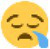, 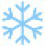, 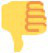, 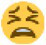, 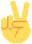, 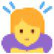, and 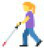. These emojis are categorized by type in Figure 2.

Figure 2 shows the types of emojis used by male and female users in Arabic tweets. It is clear from this figure that they shared similarities in terms of emoji type: Both tend to use emoji for facial expressions, hand gestures, hearts, flowers, people, and other symbols and objects in their tweets. Female users used emojis related to facial expressions, hearts, symbols, and objects more than males. Both males and females used emojis related to hand gestures, flowers, and people with only slight differences in frequency.

**Figure 3:**
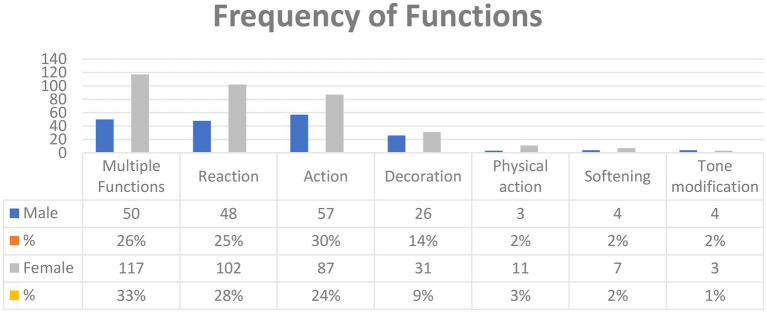
The frequency of functions in Arabic tweets.

Figure 3 shows the pragmatic functions for which emoji are used in Arabic tweets by both male and female users and their frequency. There were only seven pragmatic functions of emojis that emerged from Arabic tweets corpus which are similar to Herring and Dainas (2017). It is clear from Figure 3 that *Multiple functions* was used the most in our corpus (male, 50; female, 117), followed by *Reaction* (male, 48; female, 102), *Action* (male, 57; female, 87), *decoration* (male, 26; female, 31), *physical action* (male, 3; female, 11), *softening* (male, 4; female, 7), and *tone modification* (male, 4; female, 3). Despite the fact that there were 439 instances of 102 emojis collected, we could not find an emoji with the functions of *riff*, *mention*, or *sequence* in the corpus due to the nature of Twitter platform because the collected tweets were comments to hashtags, not chats as on other platforms.

**Figure 4:**
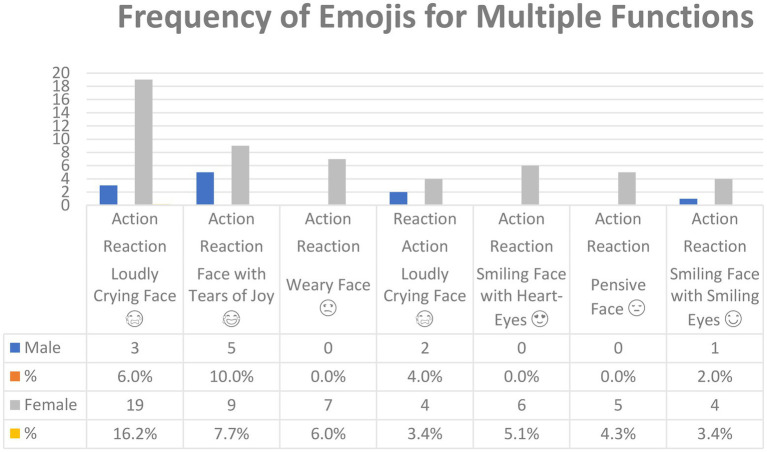
The most common emojis used for multiple functions in Arabic tweets.

Figure 4 shows the emojis used for *Multiple functions* in the corpus and their frequency. In all, 81 emojis were used for *Multiple functions*, but only those used five or more times are included in Figure 4 (see Appendix B for the lower-frequency emoji). However, the same emoji was used for different functions. For example, *Loudly Crying Face* was used for *Action-Reaction* as well as for Reaction-Action based on the possibility of function one and function two (See Appendix B).

**Figure 5:**
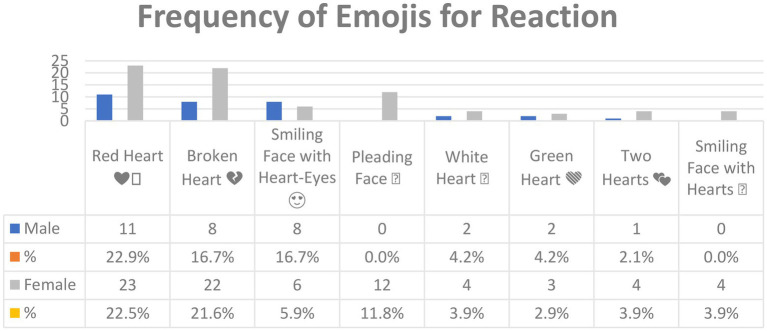
The most common emojis used for *Reaction* in Arabic tweets.

Figure 5 shows the emojis used for *Reaction* in Arabic tweets and their frequency. Note that while a total of 34 emojis were used for *Reaction*, only those with a frequency of four or more are included in Figure 5 (see Appendix C for the lower-frequency emoji). Thus, a total of eight emoji appear in Figure 5 above.

**Figure 6:**
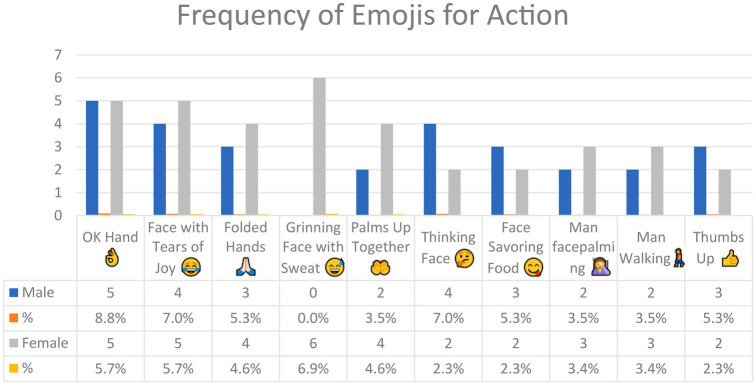
The most common emojis used for *Action* in Arabic tweets.

Figure 6 shows the emojis used for *Action* in Arabic tweets and their frequency. In all, 57 emojis were used for action, but only those with a frequency of five or more are included in Figure 6 (see Appendix D for the lower-frequency emoji). There is thus a total of 10 emojis in Figure 6 above.

**Figure 7:**
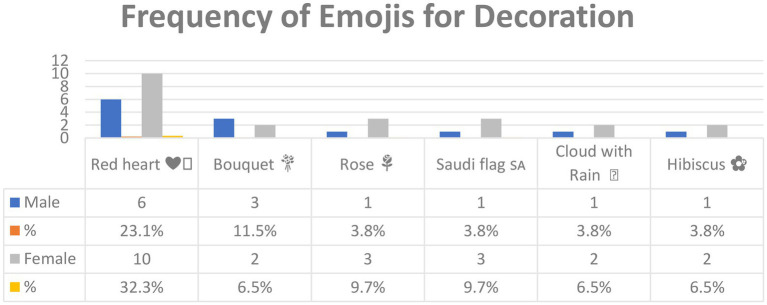
The most common emojis used for *Decoration* in Arabic tweets.

Figure 7 shows the emojis used for *Decoration* and their frequencies. In all, 24 emoji were used for Decoration, but only those with a frequency of three or more are included in Figure 7 (see Appendix E for the lower-frequency emoji). There are thus six emojis in Figure 7.

**Figure 8:**
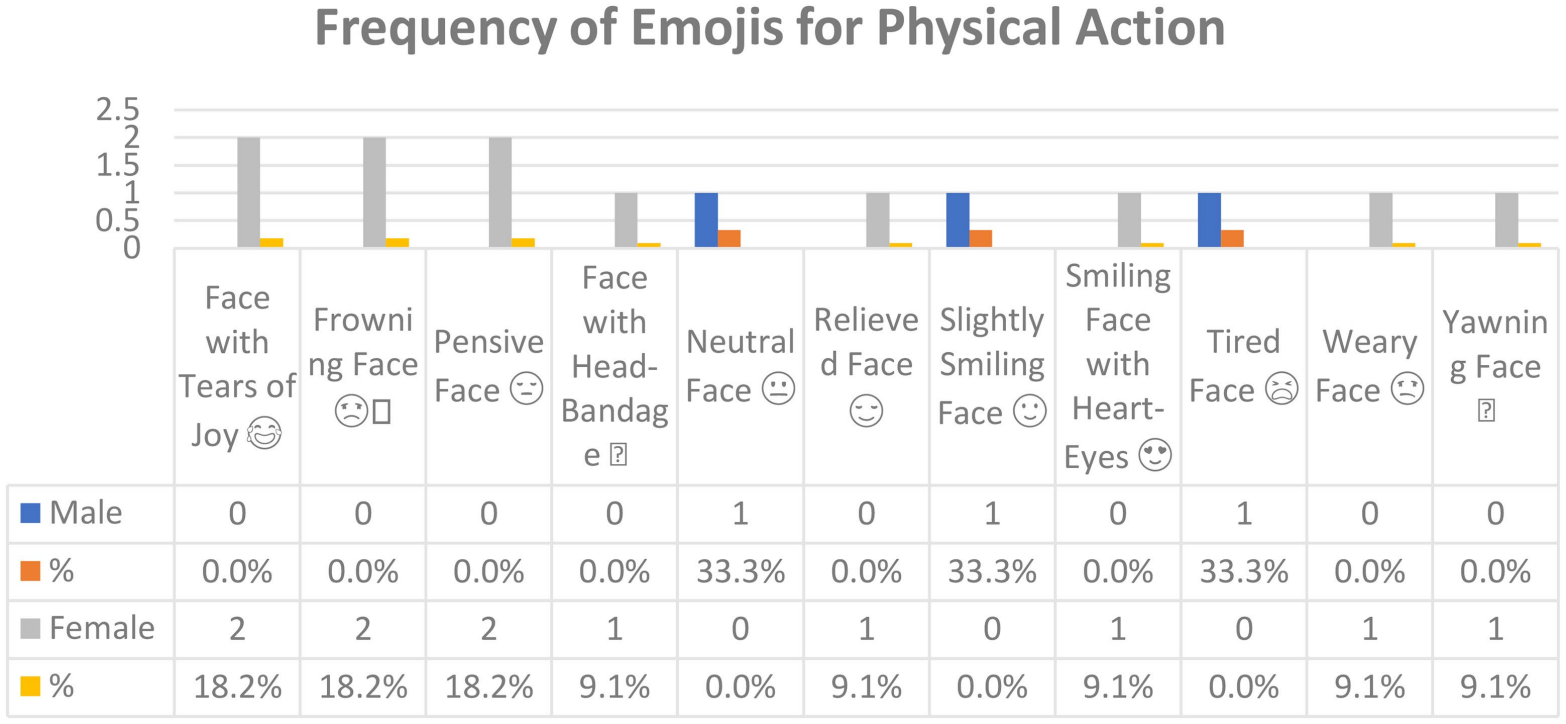
The emojis used for *Physical action* in Arabic tweets.

Figure 8 shows the emojis used for *Physical action* in Arabic tweets and their frequencies. In all, 11 emojis that were used for this function.

**Figure 9:**
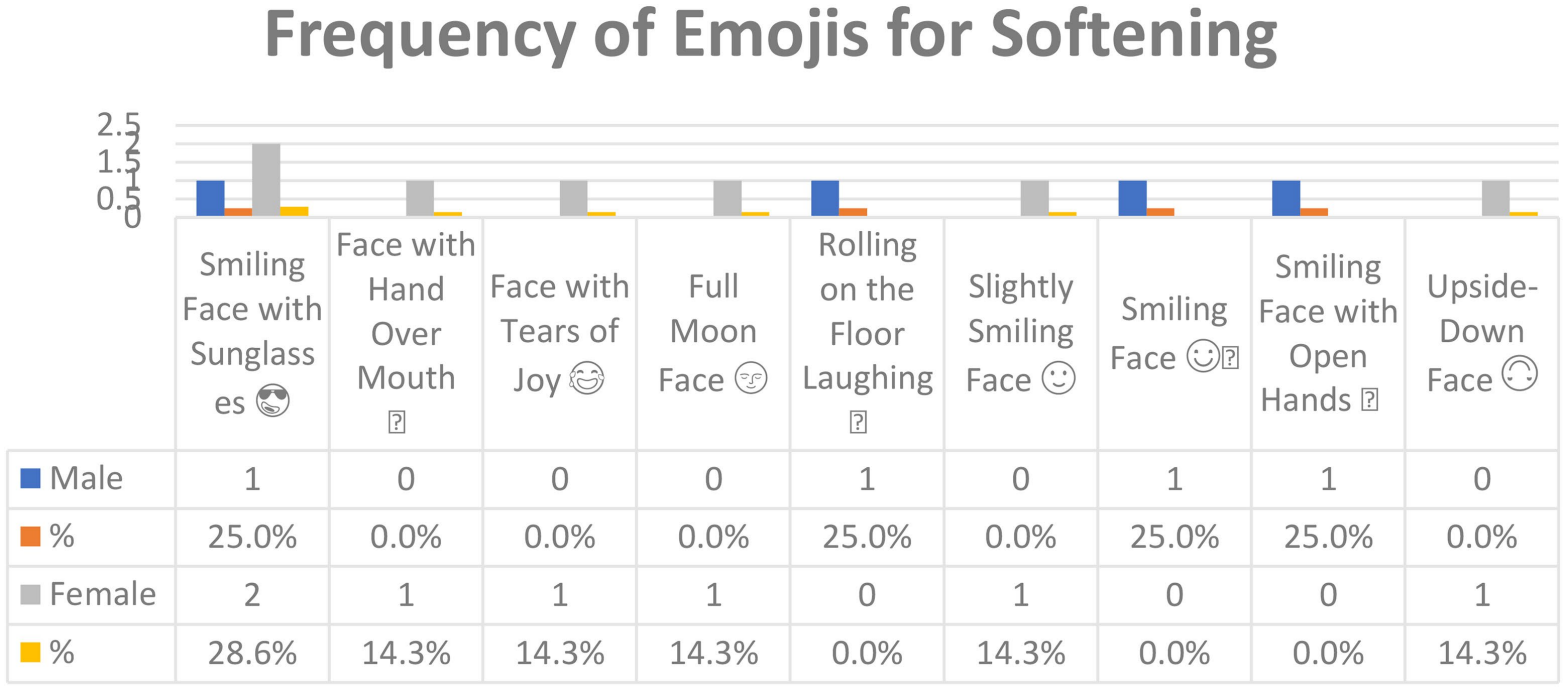
The emojis used for *Softening* in Arabic tweets.

Figure 9 shows the emojis used for *Softening* in Arabic tweets and their frequencies. In all, nine emojis were used for this function.

**Figure 10:**
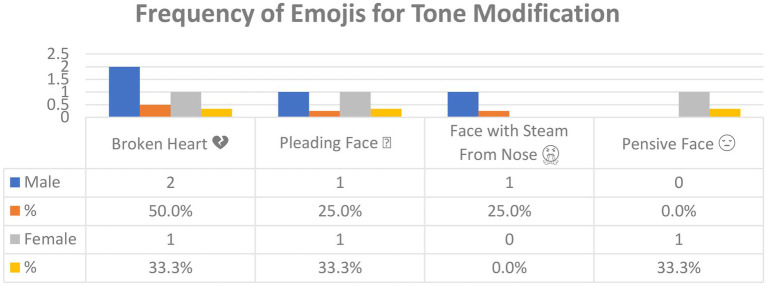
The emojis used for tone modification in Arabic tweets.

Figure 10 shows the emojis used for *tone modification* in Arabic tweets and their frequency. In all, four emojis were used for this function. *Broken Heart* was used twice by male users and once by females, followed by *Pleading Face* (male, 1; female, 1), *Face with Steam From Nose* (male, 1; female, 0), and *Pensive Face* (male, 0; female, 1).

**Figure 11:**
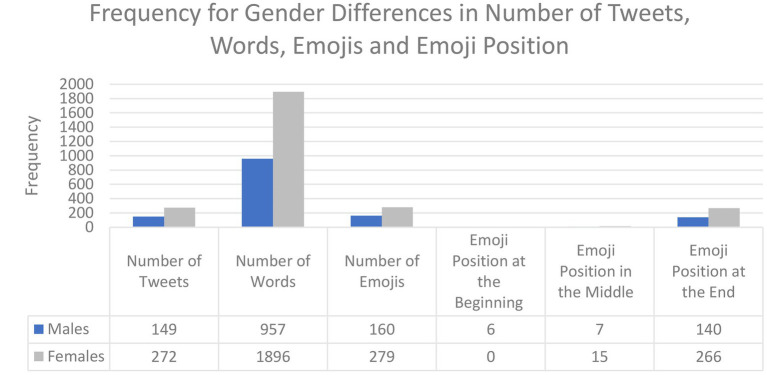
Gender differences in the number of tweets, frequency of words and emojis, and emoji position in Arabic Tweets.

Figure 11 suggests possible differences between male and female users in Arabic tweets in terms of the number of words in tweets, emojis, and emoji position. Although there is a difference between the number of the tweets collected for this study by gender, it is clear from this figure that there is no great difference in the number of words used per tweet: 6.42 words for males and 6.97 words for females. In terms of emoji use, more emojis were collected from women than men (279 versus 160, respectively), but only a slight difference in the number of emojis per tweet: males, 1.07 versus females, 1.02. In terms of emoji repetition; women repeated emojis more than men did: 52 times versus 26 times, respectively. In terms of emoji position, men used emojis at the end of their tweets 140 times, in the middle 7 times, and at the beginning 6 times, while women used emojis at the end of their tweets 266 times, in the middle 15 times, and never at the beginning.

## 6. Discussion

The main purpose of this study was to identify the common emojis used in Arabic tweets and analyze the pragmatic functions of these emojis, examine emoji position within the tweet, and identify possible gender differences. Regarding the common emojis used by Arabs on Twitter, it was obvious that *Loudly Crying Face* (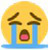), *Red Heart* (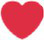), *Face with Tears of Joy* (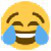), *Broken Heart* (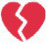), *Smiling Face with Heart-Eyes* (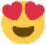), *Pleading Face* (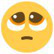), *Slightly Smiling Face* (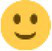), *Pensive Face* (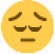), and *Weary Face* (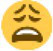) were used the most in Arabic tweets. We noted that these emojis are used to denote emotions. The results here were consistent with those of prior studies regarding the common emojis in different languages and platforms (Sampietro, 2019; Tantawi and Rosson, 2019; Wirza et al., 2019; Al-Garaady and Mahyoob, 2021; Etman and Elkareh, 2021). The results also indicated that Arab users tend to use different types of emoji, including facial expressions and hand gestures, to express their emotions and feelings in online communication, consistent with the findings of numerous studies. Moreover, it should be noted that Arab users tended to use emojis at the end of their tweets more widely, not only to end their tweets and to replace the punctuation marks, but also to indicate the functions they need to express in the context of that tweet. Additionally, there were few times where emojis were used at the beginning of the tweets, not only to prepare for the comments, but also to show the function that the user wants to convey in the tweet.

Seven pragmatic functions were found in Arabic tweets, similar to the arguments of Herring and Dainas (2017): *Multiple functions*, *Reaction*, *Action*, *Decoration*, *Physical action*, *Softening*, and *Tone modification. Mention*, *Riff*, and *Sequence* were not found in Arabic tweets, contrary to Dolot and Opina (2021), and this difference is probably to be attributed to the nature of the Twitter platform since the collected tweets were comments from public hashtags which involve only one person in the tweet. However, *Mention*, *Riff* and *Sequence* involve more than one person in the comment. Also*, Multiple functions* was the most frequent function used due to the nature of the data collected, as they were tweets to hashtags, not messages or comments. According to Herring and Dainas (2017), *Reaction* is used to express an emotion in response to something that has been posted. However, in this study emojis were used to express an attitude, not only emotions, in response to something that has been posted. Also, softening was used to make the comment more polite. However, it was found that emojis were used to soften requests as well as comments.

Consistent with Alshboul and Rababah (2021), some emojis were used for referential functions like *Kaaba* (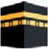), and, as reported by Yüce et al. (2021), emojis were to depict the color of the sport teams like *Blue Heart* (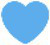) and *Yellow Heart* (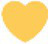) as a means to communicate with their fans and show interest and enthusiasm. According to Siyang (2018), every country has a unique culture and differences, and this cultural diversity can be seen in some emojis used in Arabic tweets like Kaaba (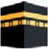), *Blue Heart* (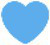), and *Yellow Heart* (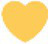).

Regarding possible gender differences, the results showed that females tend to use emojis and repeat them more than males, consistent with Herring and Dainas (2018), Al-Garaady and Mahyoob (2021), and Alshboul and Rababah (2021), but contrary to Imran et al. (2020) and Herring and Dainas (2020). Also, it was found that gender affects emoji use as *Loudly Crying Face* was the emoji used most by the women studied, while *Face with Tears of Joy* was the one used most by males. In addition, certain emojis were used by males only and vice versa, consistent with Wirza et al. (2019), Al Rashdi (2018), and Al-Rawi et al. (2020). Moreover, men tend to use emojis more widely at different positions in their tweets, while women never use them at the beginning of their tweets. Furthermore, the women used facial expressions, hearts, and symbol emojis more than men did. Finally, it was found that *Multiple functions*, *Reaction*, and *Physical action* were used by women more than men, while *Action*, *Decoration*, and *Tone modification* were used by men more than women. In contrast, *Softening* was used by men and women equally.

## 7. Conclusion

This study has focused on the common emojis used in Arabic tweets, their pragmatic functions and positions, and possible gender-based differences and similarities through a mixed qualitative and quantitative method. This section summarizes the findings and offers recommendations. This study found *Loudly Crying Face* (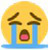), *Red Heart* (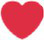), *Face with Tears of Joy* (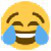), *Broken Heart* (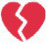), *Smiling Face with Heart-Eyes* (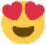), Pleading Face (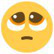), *Slightly Smiling Face* (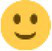), *Pensive Face* (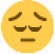), and *Weary Face* (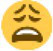) to be the common emojis used by Arab users in Twitter. Also, the emojis used in Arabic tweets have pragmatic functions similar to those identified by Herring and Dainas (2017). Only seven pragmatic functions were found, however: *Multiple functions*, *Reaction*, *Action*, *Decoration*, *Physical action*, *Softening*, and *Tone modification. Mention*, *Riff*, and *Sequence* were not found in Arabic tweets. With regard to *Tone modification*, it was the least frequent, which is inconsistent with Herring and Dainas (2020), Dolot and Opina (2021), and Dainas and Herring (2021). Also, it was found that the emojis used for Reaction derived from hearts, while the emojis used for Action were drawn from hand gestures or people, unlike Herring and Dainas (2018). In addition, the emojis used for *Physical action* and *Multiple functions* were from facial expressions, unlike Herring and Dainas (2018). Regarding gender differences, there were slight differences between men and women in terms of emoji use and repetition. Also, there were certain emojis used only by men, and vice versa. This highlight the importance of context in studying emojis, as noted in previous studies of the role of context in understanding the functions of emojis. Emojis are extremely dependent on context and may give different results within context.

The results of this study have implications for the field of Natural Language Processing in terms of creating a computer programming language that is able to identify and classify the functions of emojis based on the context of tweet in Arabic, which is a challenging task. Also, the results have implications for the designers of emojis to create a solution for the emojis that can be interpreted for two different functions as found in Loudly Crying Face. It was interpreted for action and reaction. This study has some limitations and suggests recommendations for future research. Since this study focuses on emojis, there are some limitations that should be acknowledged. The number of collected tweets and emojis is limited to generalize the findings to another platform. This study includes only 102 distinct emojis, so future studies should explore a wider range of emojis and their functions, not only on Twitter but also other platforms like WhatsApp, Instagram, or YouTube. Also, this study investigated the functions of emojis from the researchers’ perspective; future studies should investigate the functions of emojis from the users’ perspective using interviews or surveys to explore not only gender differences but also age differences. Finally, the use of stickers or avatars needs to be studied.

## Author’s note

All emojis/emoticons have been reproduced from https://www.twemoji.twitter.com and are licensed under CC-BY 4.0.

## Data availability statement

The original contributions presented in the study are included in the article/Supplementary material, further inquiries can be directed to the corresponding author.

## Author contributions

AA and MM contributed to conception and design of the study. AA organized the database, wrote the introduction, literature review, and discussion with conclusion of the manuscript. MM performed the statistical analysis, wrote the methodology, and the analysis of the manuscript. All authors contributed to the article and approved the submitted version.

## Conflict of interest

The authors declare that the research was conducted in the absence of any commercial or financial relationships that could be construed as a potential conflict of interest.

## Publisher’s note

All claims expressed in this article are solely those of the authors and do not necessarily represent those of their affiliated organizations, or those of the publisher, the editors and the reviewers. Any product that may be evaluated in this article, or claim that may be made by its manufacturer, is not guaranteed or endorsed by the publisher.
