# Supplementary material for: The pragmatic functions of emojis in Arabic tweets
Source: Front Psychol. 2023 Feb 27;13:1059672. doi: 10.3389/fpsyg.2022.1059672 (PMC10010193; doi:10.3389/fpsyg.2022.1059672)
Supplement: Supplementary file 1 [file Data_Sheet_1.pdf]

Appendix A: The emojis used in Arabic tweets 1

#	Emojis	Male	%	Female	%	Total	%
1	Loudly Crying Face 😭	5	3.1%	26	9.3%	31	7.1%
2	Red Heart ❤️	12	7.5%	17	6.1%	29	6.6%
3	Face with Tears of Joy 😂	13	8.1%	14	5.0%	27	6.2%
4	Broken Heart 💔	8	5.0%	14	5.0%	22	5.0%
5	Smiling Face with Heart-Eyes 😍	8	5.0%	14	5.0%	22	5.0%
6	Pleading Face 🥺	0	0.0%	18	6.5%	18	4.1%
7	Slightly Smiling Face 🙂	5	3.1%	11	3.9%	16	3.6%
8	Pensive Face 😔	0	0.0%	12	4.3%	12	2.7%
9	Weary Face 😩	1	0.6%	11	3.9%	12	2.7%
10	Relieved Face 😌	2	1.3%	6	2.2%	8	1.8%
11	Smiling Face with Smiling Eyes 😊	4	2.5%	4	1.4%	8	1.8%
12	Grinning Face with Sweat 😅	0	0.0%	7	2.5%	7	1.6%
13	Rolling on the Floor Laughing 🤣	6	3.8%	1	0.4%	7	1.6%
14	Beaming Face with Smiling Eyes 😁	3	1.9%	3	1.1%	6	1.4%
15	Crying Face 😢	1	0.6%	5	1.8%	6	1.4%
16	Man Facepalming 🤦‍♂️	3	1.9%	3	1.1%	6	1.4%
17	Smiling Face with Hearts 🥰	0	0.0%	6	2.2%	6	1.4%
18	Thinking Face 🤔	4	2.5%	2	0.7%	6	1.4%
19	Thumbs Up 👍	4	2.5%	2	0.7%	6	1.4%
20	White Heart 🤍	2	1.3%	4	1.4%	6	1.4%
21	Face Savoring Food 😋	3	1.9%	2	0.7%	5	1.1%
22	Face with Hand Over Mouth 🤭	1	0.6%	4	1.4%	5	1.1%
23	Folded Hands 🙏	3	1.9%	2	0.7%	5	1.1%
24	OK Hand 👌	4	2.5%	1	0.4%	5	1.1%
25	Smiling Face ☺️	4	2.5%	1	0.4%	5	1.1%
26	Smiling Face with Sunglasses 😎	2	1.3%	3	1.1%	5	1.1%
27	Bouquet 💐	2	1.3%	2	0.7%	4	0.9%
28	Face with Head-Bandage 🤕	1	0.6%	3	1.1%	4	0.9%
29	Man Bowing 🙇‍♂️	0	0.0%	4	1.4%	4	0.9%
30	Man Walking 🚶‍♂️	2	1.3%	2	0.7%	4	0.9%
31	Palms Up Together 🤲	2	1.3%	2	0.7%	4	0.9%
32	Raised Hand ✋	1	0.6%	3	1.1%	4	0.9%
33	Rose 🌹	1	0.6%	3	1.1%	4	0.9%
34	Sleeping Face 😴	3	1.9%	1	0.4%	4	0.9%
35	Smiling Face with Open Hands 🤗	3	1.9%	1	0.4%	4	0.9%
36	Two Hearts 💕	1	0.6%	3	1.1%	4	0.9%
37	Unamused Face 😒	1	0.6%	3	1.1%	4	0.9%
38	Upside-Down Face 🙃	1	0.6%	3	1.1%	4	0.9%
39	Cloud with Rain 🌧️	1	0.6%	2	0.7%	3	0.7%
40	Face Without Mouth 😶	1	0.6%	2	0.7%	3	0.7%
41	Flexed Biceps 💪	0	0.0%	3	1.1%	3	0.7%

42	Green Heart 💚	2	1.3%	1	0.4%	3	0.7%
43	Grinning Face 😃	1	0.6%	2	0.7%	3	0.7%
44	Man Running 🏃‍♂️	1	0.6%	2	0.7%	3	0.7%
45	New Moon Face 🌚	1	0.6%	2	0.7%	3	0.7%
46	Star-Struck 🤩	1	0.6%	2	0.7%	3	0.7%
47	Winking Face with Tongue 😜	1	0.6%	2	0.7%	3	0.7%
48	Woman Dancing 💃	0	0.0%	3	1.1%	3	0.7%
49	Yawning Face 🥱	0	0.0%	3	1.1%	3	0.7%
50	Blue Heart 💙	2	1.3%	0	0.0%	2	0.5%
51	Clapping Hands 👏	2	1.3%	0	0.0%	2	0.5%
52	Drooling Face 🤤	2	1.3%	0	0.0%	2	0.5%
53	Enraged Face 😡	0	0.0%	2	0.7%	2	0.5%
54	Four Leaf Clover 🍀	1	0.6%	1	0.4%	2	0.5%
55	Frowning Face ☹️	0	0.0%	2	0.7%	2	0.5%
56	Full Moon Face 🌝	0	0.0%	2	0.7%	2	0.5%
57	Hibiscus 🌺	1	0.6%	1	0.4%	2	0.5%
58	Neutral Face 😐	2	1.3%	0	0.0%	2	0.5%
59	Persevering Face 😣	1	0.6%	1	0.4%	2	0.5%
60	Person Shrugging 🤷	1	0.6%	1	0.4%	2	0.5%
61	Saudi flag SA 🇸🇦	1	0.6%	1	0.4%	2	0.5%
62	Waving Hand 👋	2	1.3%	0	0.0%	2	0.5%
63	Zany Face 🤪	1	0.6%	1	0.4%	2	0.5%
64	Index Pointing Up ☝️	0	0.0%	1	0.4%	1	0.2%
65	Anxious face with sweat 😰	0	0.0%	1	0.4%	1	0.2%
66	Backhand Index Pointing Down 👇	1	0.6%	0	0.0%	1	0.2%
67	Cherry Blossom 🌸	0	0.0%	1	0.4%	1	0.2%
68	Crossed Fingers 🤞	1	0.6%	0	0.0%	1	0.2%
69	Crown 👑	1	0.6%	0	0.0%	1	0.2%
70	Disappointed Face 😞	0	0.0%	1	0.4%	1	0.2%
71	Dove 🕊️	0	0.0%	1	0.4%	1	0.2%
72	Exploding Head 🤯	1	0.6%	0	0.0%	1	0.2%
73	Eyes 👀	0	0.0%	1	0.4%	1	0.2%
74	Face Blowing a Kiss 😘	0	0.0%	1	0.4%	1	0.2%
75	Face Screaming in Fear 😱	1	0.6%	0	0.0%	1	0.2%
76	Face with Rolling Eyes 🙄	0	0.0%	1	0.4%	1	0.2%
77	Face with Steam From Nose 😤	1	0.6%	0	0.0%	1	0.2%
78	Face with tongue 😛	0	0.0%	1	0.4%	1	0.2%
79	Fire 🔥	0	0.0%	1	0.4%	1	0.2%
80	Grinning Face with Smiling Eyes 😄	1	0.6%	0	0.0%	1	0.2%
81	Growing Heart 💗	1	0.6%	0	0.0%	1	0.2%
82	Handshake 🤝	1	0.6%	0	0.0%	1	0.2%
83	Herb 🌿	1	0.6%	0	0.0%	1	0.2%
84	Kaaba 🕋	1	0.6%	0	0.0%	1	0.2%
85	Kissing Face with Closed Eyes 😚	1	0.6%	0	0.0%	1	0.2%
86	Man Raising Hand 🙋‍♂️	1	0.6%	0	0.0%	1	0.2%
87	Oncoming Fist 👊	1	0.6%	0	0.0%	1	0.2%

88	Orange Heart 🧡	0	0.0%	1	0.4%	1	0.2%
89	Pinching Hand 🤏	0	0.0%	1	0.4%	1	0.2%
90	Purple Heart 💜	0	0.0%	1	0.4%	1	0.2%
91	Revolving Hearts 💞	1	0.6%	0	0.0%	1	0.2%
92	Shamrock ☘️	1	0.6%	0	0.0%	1	0.2%
93	Sleepy Face 😪	0	0.0%	1	0.4%	1	0.2%
94	Smiling Face with Halo 😇	1	0.6%	0	0.0%	1	0.2%
95	Snowflake ❄️	0	0.0%	1	0.4%	1	0.2%
96	Thumbs Down 👎	0	0.0%	1	0.4%	1	0.2%
97	Tired Face 😫	0	0.0%	1	0.4%	1	0.2%
98	Victory Hand ✌️	0	0.0%	1	0.4%	1	0.2%
99	Woman Bowing 🙇‍♀️	0	0.0%	1	0.4%	1	0.2%
100	Woman with White Cane 👩‍🦯	0	0.0%	1	0.4%	1	0.2%
101	Writing Hand ✍️	1	0.6%	0	0.0%	1	0.2%
102	Yellow Heart 💛	1	0.6%	0	0.0%	1	0.2%
	Total	160	100.0%	279	100.0%	439	100.0%

Appendix B: The emojis used for multiple functions in Arabic tweets

Emoji	Function #1	Function #2	Function #3	Male	%	Female	%	Grand Total	%
Loudly Crying Face 😭	Reaction	Action		3	6.0%	19	16.2%	22	13.2%
Face with Tears of Joy 😂	Reaction	Action		5	10.0%	9	7.7%	14	8.4%
Weary Face 😩	Reaction	Action		0	0.0%	7	6.0%	7	4.2%
Loudly Crying Face 😭	Action	Reaction		2	4.0%	4	3.4%	6	3.6%
Smiling Face with Heart-Eyes 😍	Reaction	Action		0	0.0%	6	5.1%	6	3.6%
Pensive Face 😔	Reaction	Action		0	0.0%	5	4.3%	5	3.0%
Smiling Face with Smiling Eyes 😊	Reaction	Action		1	2.0%	4	3.4%	5	3.0%
Beaming Face with Smiling Eyes 😁	Reaction	Action		1	2.0%	3	2.6%	4	2.4%
Pleading Face 🥺	Reaction	Action			0.0%	4	3.4%	4	2.4%
Relieved Face 😌	Reaction	Action		1	2.0%	3	2.6%	4	2.4%
Rolling on the Floor Laughing 🤣	Reaction	Action		4	8.0%	0	0.0%	4	2.4%
Face with Tears of Joy 😂	Action	Reaction		0	0.0%	3	2.6%	3	1.8%
Slightly Smiling Face 🙂	Action	Reaction		1	2.0%	2	1.7%	3	1.8%
Broken Heart 💔	Tone modification	Reaction		0	0.0%	2	1.7%	2	1.2%
Crying Face 😢	Action	Reaction		1	2.0%	1	0.9%	2	1.2%
Crying Face 😢	Reaction	Action		0	0.0%	2	1.7%	2	1.2%
Grinning Face 😃	Reaction	Action		0	0.0%	2	1.7%	2	1.2%

Pensive Face 😔	Physical action	Reaction		0	0.0%	2	1.7%	2	1.2%
Pensive Face 😔	Tone modification	Reaction	Action	0	0.0%	2	1.7%	2	1.2%
Pleading Face 🥺	Tone modification	Reaction	Action	0	0.0%	2	1.7%	2	1.2%
Slightly Smiling Face 🙂	Reaction	Action		0	0.0%	2	1.7%	2	1.2%
Slightly Smiling Face 🙂	Softening	Reaction	Action	1	2.0%	1	0.9%	2	1.2%
Star-Struck 🤩	Reaction	Action		1	2.0%	1	0.9%	2	1.2%
Unamused Face 😒	Action	Reaction		1	2.0%	1	0.9%	2	1.2%
Upside-Down Face 🙃	Reaction	Action		1	2.0%	1	0.9%	2	1.2%
Beaming Face with Smiling Eyes 😁	Softening	Reaction	Action	1	2.0%	0	0.0%	1	0.6%
Broken Heart 💔	Physical action	Reaction		0	0.0%	1	0.9%	1	0.6%
Clapping Hands 👏	Reaction	Action		1	2.0%	0	0.0%	1	0.6%
Drooling Face 🤤	Action	Reaction		1	2.0%		0.0%	1	0.6%
Drooling Face 🤤	Physical action	Reaction		1	2.0%	0	0.0%	1	0.6%
Enraged Face 😡	Action	Reaction		0	0.0%	2	1.7%	2	1.2%
Face Blowing a Kiss 😘	Action	Reaction		0	0.0%	1	0.9%	1	0.6%
Face Savoring Food 😋	Reaction	Action		1	2.0%	0	0.0%	1	0.6%
Face Screaming in Fear 😱	Action	Reaction		1	2.0%	0	0.0%	1	0.6%
Face with Hand Over Mouth 🤭	Reaction	Action		0	0.0%	1	0.9%	1	0.6%
Face with Head-Bandage 🤕	Action	Reaction		0	0.0%	1	0.9%	1	0.6%
Face with Tears of Joy 😂	Action	Softening		1	2.0%	0	0.0%	1	0.6%

Face with Tears of Joy 😂	Reaction	Action	Softening	1	2.0%	0	0.0%	1	0.6%
Face with Tears of Joy 😂	Reaction	Softening		1	2.0%	0	0.0%	1	0.6%
Face with Tears of Joy 😂	Softening	Reaction	Action	1	2.0%	0	0.0%	1	0.6%
Face with Tears of Joy 😂	Softening	Action		1	2.0%	0	0.0%	1	0.6%
Face with tongue 😛	Softening	Action		0	0.0%	1	0.9%	1	0.6%
Face Without Mouth 😶	Softening	Reaction		0	0.0%	1	0.9%	1	0.6%
Grinning Face 😀	Action	Reaction		1	2.0%		0.0%	1	0.6%
Grinning Face with Sweat 😅	Reaction	Action		0	0.0%	1	0.9%	1	0.6%
Kissing Face with Closed Eyes 😚	Reaction	Action		1	2.0%	0	0.0%	1	0.6%
Loudly Crying Face 😭	Tone modification	Reaction	Action	0	0.0%	1	0.9%	1	0.6%
Man Bowing 🙇‍♂️	Reaction	Action		0	0.0%	1	0.9%	1	0.6%
Man Facepalming 🤦‍♂️	Reaction	Action		1	2.0%	0	0.0%	1	0.6%
Man Walking 🚶‍♂️	Action	Reaction		0	0.0%	1	0.9%	1	0.6%
New Moon Face 🌚	Reaction	Action		1	2.0%	0	0.0%	1	0.6%
Pensive Face 😔	Reaction	Softening		0	0.0%	1	0.9%	1	0.6%
Persevering Face 😣	Reaction	Action		1	2.0%	0	0.0%	1	0.6%
Pleading Face 🥺	Physical action	Reaction		0	0.0%	1	0.9%	1	0.6%
Pleading Face 🥺	Reaction	Action	Softening	0	0.0%	1	0.9%	1	0.6%
Pleading Face 🥺	Tone modification	Reaction		0	0.0%	1	0.9%	1	0.6%
Relieved Face 😌	Reaction	Softening		1	2.0%	0	0.0%	1	0.6%
Rolling on the Floor	Action	Reaction		1	2.0%	0	0.0%	1	0.6%

Laughing 🤣									
Sleeping Face 😴	Reaction	Action		1	2.0%	0	0.0%	1	0.6%
Sleepy Face 😪	Reaction	Action		0	0.0%	1	0.9%	1	0.6%
Slightly Smiling Face 🙂	Action	Softening		1	2.0%	0	0.0%	1	0.6%
Slightly Smiling Face 🙂	Reaction	Action	Softening	1	2.0%	0	0.0%	1	0.6%
Slightly Smiling Face 🙂	Softening	Reaction		0	0.0%	1	0.9%	1	0.6%
Slightly Smiling Face 🙂	Softening	Action	Reaction	0	0.0%	1	0.9%	1	0.6%
Smiling Face ☺️	Reaction	Action		1	2.0%	0	0.0%	1	0.6%
Smiling face ☺️	Action	Softening		0	0.0%	1	0.9%	1	0.6%
Smiling Face with Heart-Eyes 😍	Physical action	Reaction		0	0.0%	1	0.9%	1	0.6%
Smiling Face with Hearts 🥰	Physical action	Reaction		0	0.0%	1	0.9%	1	0.6%
Smiling Face with Hearts 🥰	Reaction	Action		0	0.0%	1	0.9%	1	0.6%
Thumbs Up 👍	Action	Reaction		1	2.0%	0	0.0%	1	0.6%
Thumbs Up 👍	Reaction	Action		1	2.0%	0	0.0%	1	0.6%
Tired Face 😫	Reaction	Action		0	0.0%	1	0.9%	1	0.6%
Unamused Face 😒	Tone modification	Reaction	Action	0	0.0%	1	0.9%	1	0.6%
Weary Face 😩	Physical action	Reaction		1	2.0%	0	0.0%	1	0.6%
Winking Face with Tongue 😜	Reaction	Action		0	0.0%	1	0.9%	1	0.6%
Winking Face with Tongue 😜	Reaction	Softening		1	2.0%	0	0.0%	1	0.6%
Winking Face with Tongue 😜	Softening	Action		0	0.0%	1	0.9%	1	0.6%

Yawning Face 🥱	Reaction	Action	0	0.0%	1	0.9%	1	0.6%
Zany Face 🤪	Action	Reaction	1	2.0%	0	0.0%	1	0.6%
Zany Face 🤪	Reaction	Physical action	0	0.0%	1	0.9%	1	0.6%
Total			50	100%	117	100.0%	167	100%

Appendix C: The emojis used for reaction in Arabic tweets

Emojis	Male	%	Female	%	Grand Total	%
Red Heart ❤️	11	22.9%	23	22.5%	34	22.7%
Broken Heart 💔	8	16.7%	22	21.6%	30	20.0%
Smiling Face with Heart-Eyes 😍	8	16.7%	6	5.9%	14	9.3%
Pleading Face 🥺	0	0.0%	12	11.8%	12	8.0%
White Heart 🤍	2	4.2%	4	3.9%	6	4.0%
Green Heart 💚	2	4.2%	3	2.9%	5	3.3%
Two Hearts 💕	1	2.1%	4	3.9%	5	3.3%
Smiling Face with Hearts 🥰	0	0.0%	4	3.9%	4	2.7%
Blue Heart 💙	2	4.2%	1	1.0%	3	2.0%
New Moon Face 🌚	0	0.0%	3	2.9%	3	2.0%
Purple Heart 💜	2	4.2%	1	1.0%	3	2.0%
Relieved Face 😌	0	0.0%	3	2.9%	3	2.0%
Sleeping Face 😴	3	6.3%	0	0.0%	3	2.0%
Smiling Face with Smiling Eyes 😊	1	2.1%	2	2.0%	3	2.0%
Face with Tears of Joy 😂	2	4.2%	0	0.0%	2	1.3%
Face Without Mouth 😶	1	2.1%	1	1.0%	2	1.3%
Weary Face 😩	0	0.0%	2	2.0%	2	1.3%
Yellow Heart 💛	1	2.1%	1	1.0%	2	1.3%
Anxious face with sweat 😰	0	0.0%	1	1.0%	1	0.7%
Disappointed Face 😞	0	0.0%	1	1.0%	1	0.7%
Drooling Face 🤤	1	2.1%	0	0.0%	1	0.7%
Fire 🔥	0	0.0%	1	1.0%	1	0.7%
Full Moon Face 🌝	0	0.0%	1	1.0%	1	0.7%
Loudly Crying Face 😭	0	0.0%	1	1.0%	1	0.7%
Pensive Face 😔	1	2.1%	0	0.0%	1	0.7%
Persevering Face 😣	0	0.0%	1	1.0%	1	0.7%
Rose 🌹	0	0.0%	1	1.0%	1	0.7%
Smiling Face with Halo 😇	1	2.1%	0	0.0%	1	0.7%
Smiling Face with Open Hands 🤗	0	0.0%	1	1.0%	1	0.7%
Smiling Face with Sunglasses 😎	1	2.1%	0	0.0%	1	0.7%
Star-Struck 🤩	0	0.0%	1	1.0%	1	0.7%
Unamused Face 😒	0	0.0%	1	1.0%	1	0.7%
Total	48	100.0%	102	100.0%	150	100.0%

Appendix D: Emojis for action in Arabic tweets

Emojis	Male	%	Female	%	Grand Total	%
OK Hand 👌	5	8.8%	5	5.7%	10	6.9%
Face with Tears of Joy 😂	4	7.0%	5	5.7%	9	6.3%
Folded Hands 🙏	3	5.3%	4	4.6%	7	4.9%
Grinning Face with Sweat 😅	0	0.0%	6	6.9%	6	4.2%
Palms Up Together 🤲	2	3.5%	4	4.6%	6	4.2%
Thinking Face 🤔	4	7.0%	2	2.3%	6	4.2%
Face Savoring Food 😋	3	5.3%	2	2.3%	5	3.5%
Man facepalming 🤦‍♂️	2	3.5%	3	3.4%	5	3.5%
Man Walking 🚶	2	3.5%	3	3.4%	5	3.5%
Thumbs Up 👍	3	5.3%	2	2.3%	5	3.5%
Man Bowing 🙇	1	1.8%	3	3.4%	4	2.8%
Raised Hand ✋	1	1.8%	3	3.4%	4	2.8%
Slightly Smiling Face 🙂	0	0.0%	4	4.6%	4	2.8%
Clapping Hands 👏	3	5.3%	0	0.0%	3	2.1%
Crying Face 😢	0	0.0%	3	3.4%	3	2.1%
Face with Hand Over Mouth 🤭	1	1.8%	2	2.3%	3	2.1%
Flexed Biceps 💪	0	0.0%	3	3.4%	3	2.1%
Loudly Crying Face 😭	0	0.0%	3	3.4%	3	2.1%
Man Running 🏃	1	1.8%	2	2.3%	3	2.1%
Woman Dancing 💃	0	0.0%	3	3.4%	3	2.1%
Face with Head-Bandage 🤕	1	1.8%	1	1.1%	2	1.4%
Handshake 🤝	1	1.8%	1	1.1%	2	1.4%
Person Shrugging 🤷	1	1.8%	1	1.1%	2	1.4%
Pleading Face 🥺	0	0.0%	2	2.3%	2	1.4%
Rolling on the Floor Laughing 🤣	0	0.0%	2	2.3%	2	1.4%
Sleeping Face 😴	0	0.0%	2	2.3%	2	1.4%
Smiling Face ☺️	2	3.5%	0	0.0%	2	1.4%
Smiling Face with Heart-Eyes 😍	1	1.8%	1	1.1%	2	1.4%
Smiling Face with Open Hands 🤗	2	3.5%	0	0.0%	2	1.4%
Smiling Face with Smiling Eyes 😊	2	3.5%	0	0.0%	2	1.4%
Waving Hand 👋	2	3.5%	0	0.0%	2	1.4%
Weary Face 😩	0	0.0%	2	2.3%	2	1.4%
Index Pointing Up ☝️	0	0.0%	1	1.1%	1	0.7%
Backhand Index Pointing Down 👇	1	1.8%	0	0.0%	1	0.7%
Beaming Face with Smiling Eyes 😁	1	1.8%	0	0.0%	1	0.7%
Broken Heart 💔	0	0.0%	1	1.1%	1	0.7%
Crossed Fingers 🤞	1	1.8%	0	0.0%	1	0.7%
Exploding Head 🤯	1	1.8%	0	0.0%	1	0.7%
Eyes 👀	0	0.0%	1	1.1%	1	0.7%
Face with Rolling Eyes 🙄	0	0.0%	1	1.1%	1	0.7%
Grinning Face 😀	1	1.8%	0	0.0%	1	0.7%

Grinning Face with Smiling Eyes 😄	1	1.8%	0	0.0%	1	0.7%
Man Raising Hand 🙋‍♂️	1	1.8%	0	0.0%	1	0.7%
Neutral Face 😐	1	1.8%	0	0.0%	1	0.7%
Oncoming Fist 👊	1	1.8%	0	0.0%	1	0.7%
Pinching Hand 🤏	0	0.0%	1	1.1%	1	0.7%
Smiling Face with Hearts 🥰	0	0.0%	1	1.1%	1	0.7%
Smiling Face with Sunglasses 😎	0	0.0%	1	1.1%	1	0.7%
Thumbs Down 👎	0	0.0%	1	1.1%	1	0.7%
Upside-Down Face 🙃	0	0.0%	1	1.1%	1	0.7%
Victory Hand ✌️	0	0.0%	1	1.1%	1	0.7%
Woman bowing 🙇‍♀️	0	0.0%	1	1.1%	1	0.7%
Woman with White Cane 👩‍🦯	0	0.0%	1	1.1%	1	0.7%
Writing Hand ✍️	1	1.8%	0	0.0%	1	0.7%
Yawning Face 🥱	0	0.0%	1	1.1%	1	0.7%
Total	57	100%	87	100.0%	144	100%

Appendix E: The emojis used for decoration in Arabic tweets

**Emojis**	**Male**	**%**	**Female**	**%**	**Grand Total**	**%**
Red heart ❤️	6	23.1%	10	32.3%	16	28.1%
Bouquet 💐	3	11.5%	2	6.5%	5	8.8%
Rose 🌹	1	3.8%	3	9.7%	4	7.0%
Saudi flag SA 🇸🇦	1	3.8%	3	9.7%	4	7.0%
Cloud with Rain 🌧️	1	3.8%	2	6.5%	3	5.3%
Hibiscus 🌺	1	3.8%	2	6.5%	3	5.3%
Blue Heart 💙	2	7.7%	0	0.0%	2	3.5%
Cherry Blossom 🌸	1	3.8%	1	3.2%	2	3.5%
Four Leaf Clover 🍀	1	3.8%	1	3.2%	2	3.5%
Growing Heart 💗	1	3.8%	1	3.2%	2	3.5%
Purple Heart 💜	1	3.8%	1	3.2%	2	3.5%
White Heart 🤍	1	3.8%	1	3.2%	2	3.5%
Crown 👑	1	3.8%	0	0.0%	1	1.8%
Dove 🕊️	0	0.0%	1	3.2%	1	1.8%
Green Heart 💚	1	3.8%	0	0.0%	1	1.8%
Herb 🌿	1	3.8%	0	0.0%	1	1.8%
Kaaba 🕋	1	3.8%	0	0.0%	1	1.8%
Orange Heart 🧡	0	0.0%	1	3.2%	1	1.8%
Revolving Hearts 💞	1	3.8%	0	0.0%	1	1.8%
Shamrock ☘️	1	3.8%	0	0.0%	1	1.8%
Snowflake ❄️	0	0.0%	1	3.2%	1	1.8%
Two Hearts 💕	0	0.0%	1	3.2%	1	1.8%
Total	26	100%	31	100.0%	57	100%
